# Influence of the Alcohol Present in a Phytotherapic Tincture on Male Rat Lipid Profiles and Renal Function

**DOI:** 10.1155/2015/762373

**Published:** 2015-12-28

**Authors:** Fernanda Coleraus Silva, Juliete Gomes de Lara de Souza, Alana Meira Reichert, Renata Prestes Antonangelo, Rodrigo Suzuki, Ana Maria Itinose, Carla Brugin Marek

**Affiliations:** ^1^Laboratory of Cellular Toxicology, State University of Western Paraná, Rua Universitária 1619, 85819110 Cascavel, PR, Brazil; ^2^Department of Veterinary Medicine, Dynamic Union of the Faculty Falls, 85852010 Foz do Iguaçu, PR, Brazil; ^3^Assistance Center in Toxicology (CEATOX), Hospital University of Western Paraná, Avenida Tancredo Neves 3224, 85806470 Cascavel, PR, Brazil

## Abstract

This study evaluated the influence of the alcohol present in a formulation of the antiophidic phytotherapic tincture, Específico-Pessôa, on rat blood biochemical and hematological parameters, and on organ histology. Three groups of rats were treated orally for 10, 15, or 30 days; one group received the tincture, the other received alcohol alone, and the third was a control group. The results of this study indicated that cholesterol levels were significantly increased after 10 days in the alcohol and tincture groups, although these decreased after 30 days in the tincture group. Triglyceride levels were significantly reduced after 15 days in the tincture group and after 30 days in the alcohol and tincture groups. A higher creatinine level was observed in the alcohol and tincture groups after 15 and 30 days. The uric acid levels in these groups were reduced at 10 and 30 days, although this metabolite was elevated at 15 days in the alcohol group. Hydropic multifocal degeneration with lymphohistiocytic infiltration and some polymorphonuclear cells was observed in the livers of rats treated with either the tincture or alcohol. These data demonstrate the importance of considering the potential actions of the alcohol present in pharmaceutical formulations.

## 1. Introduction

It is well known that the therapeutic effects of medicines can be influenced by other substances, particularly by alcohol [1]. Although some studies have shown beneficial effects of moderate alcohol consumption on the risk for cardiac disease [2], other studies had shown increased risks for liver cirrhosis, neuromuscular disorders, and cancers [3, 4]. The recreational use of alcohol usually involves the ingestion of alcoholic beverages such as beers, wines, and spirits. However, nonrecreational exposure can also occur in certain populations due to the ingestion of herbal medications containing alcohol in the formulation.

The use of alcohol is sometimes necessary to extract bioactive substances from plants [5] and it is common for traditional and folk medicines to have a high alcohol content; this may be >10%. It is possible that alcohol may interfere with the metabolism and mechanisms of action of the bioactive compounds present in herbal medicines. Some studies have investigated the pharmacokinetic and pharmacodynamic mechanisms involved in the interactions between alcohol and various drug classes [5, 6]; however few studies have investigated the influence of alcohol on the actions of compounds present in phytotherapic tinctures. This is an important issue in toxicology.

**Figure 1:**
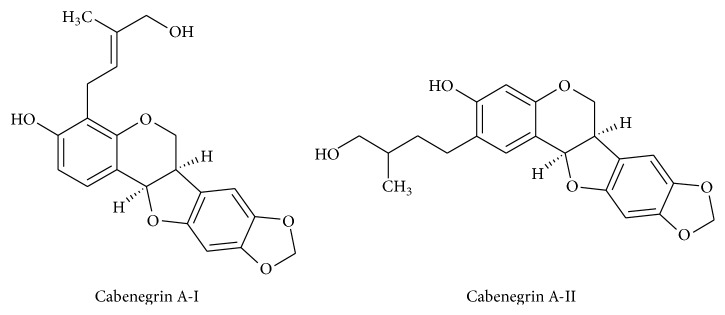
Structure of two pterocarpans isolated and identified in Específico-Pessôa phytotherapic tincture by Nakagawa et al. (1982) [7].

The phytotherapic tincture, Específico-Pessôa, has been used for more than 30 years as a supportive therapy for snake bites, particularly in the north and northeast of Brazil [7, 8]. This is a hydroalcoholic extract of the root of a plant commonly known as “cabeça-de-negro.” Four Brazilian plant species are designated as “cabeça-de-negro”:* Cayaponia tayuya* (Kell.) Cogn.,* Cayaponia espelina* Cogn.,* Annona coriacea* (Mart.), and* Wilbrandia *sp. [9]. There are reports of Específico-Pessôa tincture use as a supportive therapy and as the only form of treatment in certain regions [8]. There are few studies of the constituents of this tincture and most of these have focused on pterocarpans. The antiophidic effects of Específico-Pessôa have been suggested to arise from the actions of two pterocarpans, cabenegrin A-I and cabenegrin A-II (Figure 1), which were first isolated and identified by Nakagawa et al. (1982) [7]. Our previous research found that Específico-Pessôa altered body weight and the lipid profile in rats; some of these changes may be due to the alcohol present in the formulation [10]. Despite the widespread use of herbal medicines, there is little information in the literature about the possible pharmacodynamic effects of the alcohol present in these extracts. Therefore, the purpose of this study was to investigate the effects of the recommended dose of Específico-Pessôa on biochemical and hematological parameters at different time-points (10, 15, and 30 days) and to determine the contribution of alcohol to these effects. These times were chosen to reflect common tincture treatment periods.

## 2. Materials and Methods

### 2.1. Extract and Formulation

The phytotherapic tincture employed in the present work was purchased from a local drugstore (Cascavel, Brazil). This Específico-Pessôa was a hydroalcoholic extract manufactured in Ceará, Brazil (Register number 262, Department of Public Health of Rio de Janeiro). The usual adult dosage is 1.0 mL diluted in 14.0 mL of water, one to three times daily.

### 2.2. Animals

Adult male Wistar rats weighing 220–280 g were provided by the Central Animal Facility of the University and were fed* ad libitum* with a standard laboratory diet (Nuvilab). They were housed in propylene cages at 22 ± 2°C in a room with a 12 h light/dark cycle. The experimental protocols and procedures used in the present study were approved by the Ethics Committee of the Western Paraná State University (Cascavel, Brazil) for the care and use of laboratory animals (Approval number 17/2014-CE).

### 2.3. Alcohol Assay

The alcohol content of the phytotherapic Específico-Pessôa tincture was measured using the method described by Widmark (1964) [11]. Essentially, alcohol was oxidized using dichromate and the excess dichromate was determined iodometrically. The result was expressed as mg alcohol·mL^−1^ tincture.

### 2.4. Experimental Procedure

All animals were treated once daily by oral gavage at a dosing volume of 0.25 mL·kg^−1^ body weight for 10, 15, or 30 days. One milliliter of the phytotherapic tincture (containing 165 mg alcohol·mL^−1^) was diluted with 14 mL water to produce the concentration recommended by the guide provided with the tincture. The alcohol (ethyl alcohol, 99.9% pure) was supplied by Merck and was diluted in water to match the concentration present in the tincture. The animals were randomly allocated to nine experimental groups with five animals in each:

 [Con10], [Con15], and [Con30] groups received normal water for 10, 15, and 30 days, respectively.
 [EP10] received 0.25 mL·kg^−1^ body weight of phytotherapic tincture for 10 days.
 [EP15] received 0.25 mL·kg^−1^ body weight of phytotherapic tincture for 15 days.
 [EP30] received 0.25 mL·kg^−1^ body weight of phytotherapic tincture for 30 days.
 [Alc10] received 2.77 mg·kg^−1^ body weight of alcohol for 10 days.
 [Alc15] received 2.77 mg·kg^−1^ body weight of alcohol for 15 days.
 [Alc30] received 2.77 mg·kg^−1^ body weight of alcohol for 30 days.

The 2.77 mg·kg^−1^ corresponds to the amount of alcohol that is present in the phytotherapic tincture. Water and food were freely available to the animals. Their general behavior was observed at 5 min, 30 min, 1 h, 2 h, 4 h, and 6 h following gavage. The behavioral observations included changes in respiration (slow or rapid shallow breathing), locomotion, tremor (rhythmic or repetitive limb movements), convulsion, hyperexcitability, reduced activity, ataxia (difficulty in walking and jumping), piloerection, drowsiness (closing the eyelids followed by eye immobility and sometimes bowing of the head), ptosis (eyelids partly closed), drooling, diarrhea, and abdominal constrictions. The body weight of the animals was monitored daily. At the end of the study, all rats were deprived of food for 12 h, anesthetized for blood collection, and subsequently sacrificed by an overdose of ketamine + xylazine anesthesia. Their organs were carefully dissected and removed for weighing, macroscopic examination, and histopathological analysis.

### 2.5. Collection of Blood and Biochemical Determinations

Blood samples were obtained by intracardiac puncture under ketamine + xylazine. Blood samples for biochemical analyses were centrifuged at 2,500 ×g for 10 min, and the levels of uric acid (UA), albumin, cholesterol, creatinine, glucose, high-density lipoprotein- (HDL-) cholesterol, low-density lipoprotein- (LDL-) cholesterol, lactate dehydrogenase (LDH), triglycerides, and urea were measured using commercial kits from Bioliquid (Brazil). Alanine aminotransferase (ALT), aspartate aminotransferase (AST), and gamma glutamyltransferase (GGT) levels were assayed using diagnostic reagent kits supplied by Ortho-Clinical Diagnostics VITROS 5.1 FS. All assays were performed in accordance with the manufacturers' instructions and protocols.

### 2.6. Hematological Analysis

All blood samples were analyzed as described by Lewis et al. (2012) [12] to determine the fraction of whole blood composed of red blood cells (hematocrit), the number of white blood cells, and the percentage of each type of white blood cell. The total level of hemoglobin and number of blood platelets were also determined using the Abbott hematology analyzer CELL-DYN Ruby. May-Grunwald-Giemsa staining was conducted using a commercial kit from Bioliquid (Brazil).

### 2.7. Organ Weights and Histopathological Analyses

The brain, heart, liver, lungs, and kidneys of all of the animals were examined macroscopically. The organs were weighed and preserved in 10% formalin. Tissue slides were prepared and stained with hematoxylin and eosin prior to microscopic examination.

### 2.8. Statistical Analysis

Data are expressed as the mean ± the standard error of the mean (SEM). The significance of differences between treated groups and the respective controls was evaluated using one-way analysis of variance (ANOVA) followed by Dunnett's test. Statistical significance was accepted at *P* < 0.05. GraphPad Prism 3 software was used for statistical analysis.

## 3. Results

The administration of alcohol or phytotherapic tincture led to drowsiness in all rats on each treatment day; this effect was reversible and lasted for up to 4 h.

### 3.1. Treatment for 10 Days

**Figure 2:**
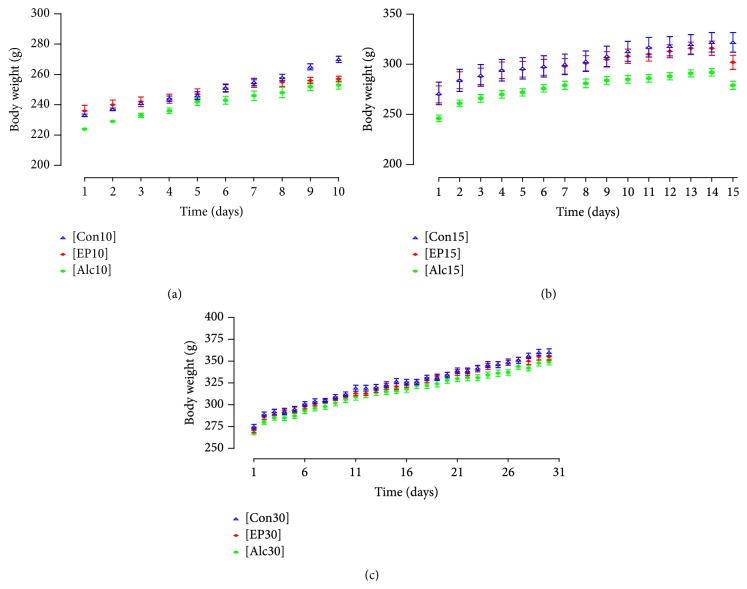
Body weights of male rats administered the indicated treatments for (a) 10 days, (b) 15 days, or (c) 30 days. Each point represents the mean ± SEM of 5 rats.

**Figure 3:**
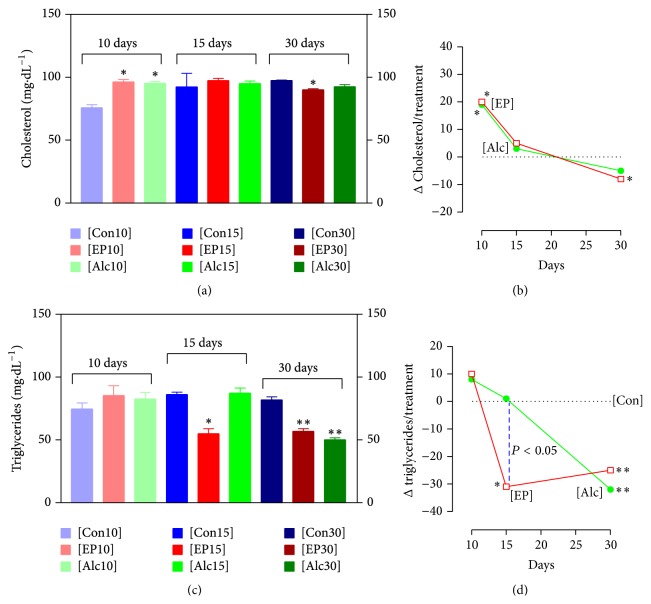
Plasma lipid profiles of male rats administered the indicated treatments for the indicated times. (a) Cholesterol levels, (b) the difference between the cholesterol level in the indicated group and that of the control group. (c) Triglyceride levels, (d) the difference between the triglyceride level in the indicated group and that of the control group. Each point represents the mean ± SEM of 5 rats. ^*∗*^ *P* < 0.05; ^*∗∗*^ *P* < 0.01, as compared to the respective control group. The *P* values refer to Dunnett's test. - - - represents a significant difference between the [EP15] and [Alc15] groups (*P* < 0.05).

**Figure 4:**
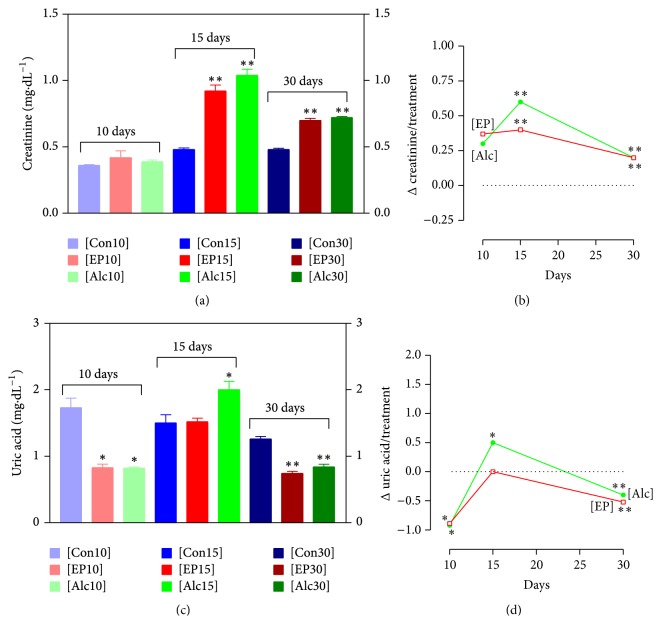
Plasma renal function measures in male rats administered the indicated treatments for the indicated times. (a) Plasma creatinine level, (b) the difference between the creatinine level in the indicated group and that of the control group. (c) Plasma uric acid level, (d) the difference between the uric acid level in the indicated group and that of the control group. Each point represents the mean ± SEM of 5 rats. ^*∗*^ *P* < 0.05; ^*∗∗*^ *P* < 0.01, as compared to the respective control group. The *P* values refer to Dunnett's test.

The [Alc10] and [EP10] groups gained less body weight than the [Con10] group, which increased from 233 ± 2.03 g to 270 ± 4.58 g. The [EP10] animals increased from 236 ± 8.03 g to 257 ± 4.16, corresponding to 56.4% of the control group weight gain; this was less than the [Alc10] group (224 ± 0.58 g to 253 ± 5.93 g, 79.8% of control), *P* < 0.05, Figure 2(a). No changes were observed in the macroscopic and histopathological analyses in these groups.  Table 1 shows that the organ weight ratios did not differ between the groups. Rats treated with alcohol or phytotherapic tincture showed an elevated cholesterol level; Figure 3(a) shows that this increased by 25% in the [Alc10] group and by 26% in the [EP10] group (*P* < 0.05 for both groups, as compared with [Con10]). The levels of triglyceride, LDL-cholesterol, and HDL-cholesterol remained unchanged in the [Alc10] and [EP10] groups, as compared with the [Con10] group (*P* > 0.05) (Figure 3(c), Table 2). Both [Alc10] and [EP10] groups showed a significantly lower UA level (*P* < 0.05; Figure 4(b)) than the [Con10] group and there was no significant difference between the [Alc10] and [EP10] groups in this respect (*P* > 0.05). The other biochemical and hematological parameters showed no significant differences between the study groups (Tables 2 and 3, resp.).

### 3.2. Treatment for 15 Days

**Figure 5:**
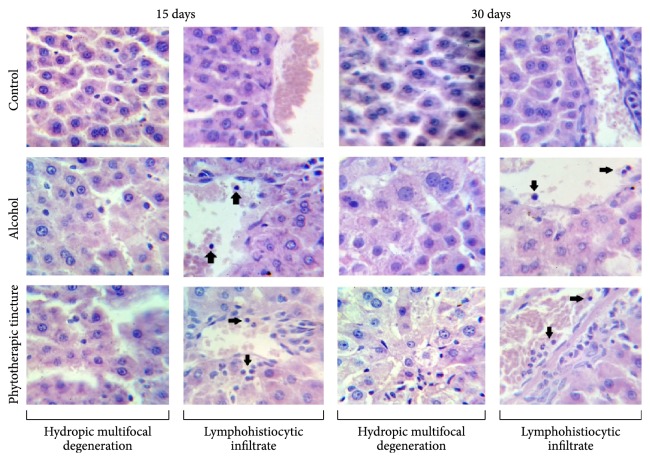
Histopathological analysis of liver tissue from rats in the indicated treatment groups. No pathological changes were observed in control rats.

The body weight gain of rats in the [EP15] group was approximately 37% lower than that observed in the [Con15] group (*P* < 0.001; Figure 2(b)). There was no difference in the body weight gain in the [EP15] (32 ± 3.69 g) and [Alc15] (33 ± 2.59 g) groups. No macroscopic alterations and/or differences in organ weight ratios were observed in the [EP15] or [Alc15] groups, as compared with [Con15] (Table 1). However, microscopic examination of the liver of rats in the [EP15] or [Alc15] groups showed hydropic multifocal degeneration with lymphohistiocytic infiltrate and some polymorphonuclear cells (Figure 5). Blood analysis (Figure 3(c)) showed a significant 36% decrease in the triglyceride level in the [EP15] group, as compared with [Con15] (*P* < 0.05), but no effect on cholesterol, LDL-cholesterol, or HDL-cholesterol levels (*P* > 0.05) (Figure 3(a), Table 2). The lipid profile was not significantly altered in the [Alc15] group. Creatinine was significantly increased in both the [EP15] and [Alc15] groups (*P* < 0.01; Figure 4(a)). There was a significant increase in the UA level of the [Alc15] group (*P* < 0.05; Figure 4(b)). The remaining biochemical parameters were not affected and the hematological parameters remained unchanged (Tables 2 and 3).

### 3.3. Treatment for 30 Days

The final body weight gain and organ weight ratios did not differ significantly between the [EP30] and [Con30] groups (Figure 2(c), Table 1). The [Alc30] group showed a significantly lower increase in body weight (*P* < 0.05). No macroscopic differences between the organs of each study group were observed. Microscopic examination of the [EP30] and [Alc30] livers showed hydropic multifocal degeneration with lymphohistiocytic infiltrate and some polymorphonuclear cells (Figure 5). The [EP30] group had significantly lower levels of cholesterol and triglyceride; these were decreased by 8% (*P* < 0.05) and 30% (*P* < 0.01), respectively, as compared with [Con30]. The [Alc30] group had a reduced triglyceride level only (*P* < 0.01; Figures 3(a) and 3(c)). The [EP30] and [Alc30] groups showed elevated creatinine (*P* < 0.01) and reduced UA (*P* < 0.01) levels (Figure 4). The other biochemical and hematological parameters remained unchanged (Tables 2 and 3).

## 4. Discussion

Although there are controversies in this area, alcohol is known to cause both acute and chronic physiological effects. Several factors may contribute to the nature and intensity of these effects, including the alcohol dose, rate of intake, and type of drink [13, 14]. While many studies have investigated different types of alcoholic beverages, the alcohol present in pharmaceutical formulations has been largely ignored. The present study showed that although pterocarpans may have beneficial effects, regular exposure to the alcohol present in this type of phytotherapic tincture may lead to serious biochemical alterations.

Drowsiness was the first effect observed and this was due to alcohol. The depressant effect of alcohol on the central nervous system is well characterized [15, 16]. Some reductions in body weight gain were observed in rats treated with alcohol and to a lesser extent in those treated with phytotherapic tincture. Interestingly, previous studies using this phytotherapic tincture in rats at a dose of 0.75 mL·kg^−1^ for 10 days led to an increase in body weight gain [10]. Alcohol-mediated effects on body weight gain have been observed by other authors and both decreases and increases have been reported, according to the amount of alcohol exposure [17, 18]. The effects of alcohol on the processes of absorption, digestion, utilization, storage, and excretion of proteins, vitamins, and minerals may partially explain these findings [19]. Furthermore, pterocarpans also affect body weight gain. A study with male C57BL/6J mice showed that pterocarpan-enriched soy leaf extract suppressed body weight gain [20]. A separate study of C57BL/6J mice treated with soy leaf extracts found a decrease in body weight gain and fat accumulation in white adipose tissue* via* several mechanisms related to adipogenesis and fat oxidation in this tissue [21]. In the present study, it is unclear why the [EP30] group showed a weight gain that was similar to that of the [Con30] group. Further studies will be required to investigate the interactions between alcohol and pterocarpans and the effects of these on weight gain.

The lipid profiles were affected differently in rats exposed to [EP] or [Alc] for different lengths of time. These findings were consistent with previous studies indicating that both alcohol [22, 23] and pterocarpans [24] affected hepatic metabolism. The effects on cholesterol levels were similar in the [EP] and [Alc] groups, while these treatments produced different effects on triglyceride levels at 15 days. The increase of cholesterol levels by approximately 20 mg·dL^−1^ in the [EP10] and [Alc10] groups may reflect increased absorption of dietary cholesterol or altered protein metabolism. Some authors have reported a relationship between alcohol and both increased cholesterol levels and protein metabolism; this reflects channeling of peripheral amino acids to hepatic protein synthesis [25] and induction of cholesterol uptake [26] by alcohol. The mechanisms involved in the reduction of cholesterol levels by pterocarpans include acyl-CoA cholesterol acyltransferase (ACAT) inhibition. The hepatic cholesteryl ester level also has a direct effect on the concentration of liver triglycerides, limiting both the mobilization and secretion of triglycerides [27]. Studies of ACAT inhibitors have shown that they influence, perhaps indirectly, triglyceride levels [27, 28]. We observed significant decreases in the triglyceride levels in the [EP15] and [EP30] groups. This reduction in the triglyceride level may have been indirectly affected by pterocarpans-mediated ACAT inhibition. However, this hypothesis requires further study since we did not measure ACAT activity or the levels of hepatic cholesteryl esters and triglycerides.

Although markers of liver function were not changed, microscopic tissue examination revealed hydropic multifocal degeneration with slight lymphohistiocytic infiltration and some polymorphonuclear cells in [EP15], [EP30], [Alc15], and [Alc30] groups. These findings suggest that although pterocarpans have been reported to have hepatoprotective effects [24, 29], alcohol toxicity may override these. Regular exposure to alcohol is associated with a variety of secondary effects, some of which relate to hepatocyte homeostasis and lipid metabolism. The observed hydropic degeneration suggested that alcohol altered cellular Na^+^ and fluid homeostasis, leading to increased intracellular water [30]. The excess reducing equivalents generated during the biotransformation of alcohol by alcohol dehydrogenase and aldehyde dehydrogenase in the liver increases the NADH/NAD^+^ ratio, affecting citrate cycle activity, and consequently reducing fatty acid oxidation. Furthermore, the increased NADH level favors fatty acid synthesis [31]. These effects promote hepatic fat accumulation. Moreover, repeat alcohol administration can also promote inflammation, as indicated by the presence of infiltrate. Interactions between alcohol and the proteins and enzymes of the hepatic interstitial tissue affect the antioxidant defense mechanism and increase generation of reactive oxygen species, which may produce an inflammatory response [32]. The accumulation of fat and cholesterol deposits [33] also reduces liver function. Some authors consider that this deterioration is progressive, starting with some lipid profile changes, followed by a potentially compensatory phase, and finally resulting in liver failure. Moreover, this progressive liver deterioration may be responsible for the disappearance of hyperlipidemia found in some cases of chronic alcohol intake [34, 35]. This could explain our blood and hepatic tissue findings in animals treated for 30 days.

In addition to its effects on the liver, alcohol alters the structure, function, regulation, and metabolism of the kidneys [36, 37]. Chronic exposure to alcohol affects renal filtration [38] and increases blood urea nitrogen and creatinine levels; some authors have recently argued that it exerts an indirect nephrotoxic effect by activating leukocytes [39]. Although this was not observed microscopically in the present study, creatinine levels were elevated in the [EP15] and [EP30] groups, suggesting possible early kidney damage, although there was no change in circulating urea levels. This renal damage may be due to the alcohol present in the phytotherapic tincture, since the [Alc] groups showed the same pattern. Another important finding that requires further study is the reduced UA levels observed in the [Alc] and [EP] groups treated for 10 and 30 days. Alcohol has previously been shown to induce hyperuricemia [40]; this effect was noted in the [Alc15] group, but not in the [EP15] group, indicating that pterocarpans attenuated the hyperuricemic effect of alcohol in rats at this time-point by an unknown mechanism. The UA level is controlled by the rate of endogenous and exogenous purine breakdown into UA and the rate of UA excretion [41]; any factor that alters liver or kidney function may influence blood UA levels [42]. Further studies could explore the mechanisms underlying the observed changes in UA levels.

## 5. Conclusion

The data presented here demonstrate that the alcohol present in a phytotherapic tincture can alter the lipid profile and renal function in rats and cause liver damage. These data have important repercussions because toxicity studies generally focus on the safety of the active ingredients; this study demonstrates the importance of considering the potential actions of the alcohol present in pharmaceutical formulations. It is noteworthy that even at the relatively low dose of 2.77 mg alcohol·kg^−1^ body weight continuous exposure to this amount of alcohol could cause significant changes in some biochemical parameters. This is important because phytotherapic tinctures are usually considered nontoxic and are used without medical supervision.

## Figures and Tables

**Table 1:** Effects of the indicated treatments on organ weight ratios in male rats.

Treatment	Group	Brain	Heart	Liver	Lung	Kidney
10 days	Control	0.667 ± 0.016	0.402 ± 0.012	3.41 ± 0.039	0.516 ± 0.020	0.845 ± 0.031
	EP	0.622 ± 0.015	0.371 ± 0.014	3.28 ± 0.163	0.545 ± 0.035	0.730 ± 0.019
	Alcohol	0.652 ± 0.012	0.398 ± 0.023	3.37 ± 0.015	0.523 ± 0.03	0.746 ± 0.018

15 days	Control	0.581 ± 0.035	0.433 ± 0.051	3.11 ± 0.118	0.565 ± 0.026	0.821 ± 0.027
	EP	0.634 ± 0.027	0.392 ± 0.018	3.45 ± 0.119	0.546 ± 0.021	0.908 ± 0.026
	Alcohol	0.672 ± 0.024	0.421 ± 0.021	3.61 ± 0.054	0.579 ± 0.026	0.916 ± 0.036

30 days	Control	0.552 ± 0.006	0.370 ± 0.007	3.03 ± 0.143	0.576 ± 0.017	0.726 ± 0.031
	EP	0.554 ± 0.014	0.372 ± 0.024	2.99 ± 0.074	0.580 ± 0.019	0.772 ± 0.012
	Alcohol	0.736 ± 0.015	0.378 ± 0.012	3.06 ± 0.062	0.579 ± 0.033	0.730 ± 0.015

EP, Específico-Pessôa phytotherapic tincture. Values represent the mean ± SEM of 5 animals.

**Table 2:** Effect of the indicated treatments on biochemical parameters in male rats.

Parameter	Control	EP	Alcohol
*10-day treatment*			
Albumin (g·dL^−1^)	2.80 ± 0.01	2.68 ± 0.09	2.70 ± 0.07
ALT (U·L^−1^)	45.6 ± 4.08	47.0 ± 6.01	48.9 ± 5.18
AST (U·L^−1^)	79.8 ± 12.30	81.5 ± 9.25	80.0 ± 13.07
Cholesterol (mg·dL^−1^)	75.8 ± 5.32	96.3 ± 3.18^*∗*^	95.3 ± 2.56^*∗*^
Creatinine (mg·dL^−1^)	0.36 ± 0.01	0.42 ± 0.09	0.39 ± 0.03
GGT (U·L^−1^)	<10	<10	<10
Glucose (mg·dL^−1^)	72 ± 13.50	80 ± 12.80	83 ± 11.90
HDL (mg·dL^−1^)	43.8 ± 3.09	54.7 ± 3.84	47.3 ± 3.17
LDL (mg·dL^−1^)	24.6 ± 2.58	30.8 ± 4.50	21.7 ± 0.88
LDH (mg·dL^−1^)	348 ± 52.30	319 ± 48.70	352 ± 31.30
Triglycerides (mg·dL^−1^)	74.5 ± 9.87	85.3 ± 13.60	82.5 ± 10.50
Urea (mg·dL^−1^)	36.3 ± 0.99	32.3 ± 5.17	36.8 ± 1.80
Uric acid (mg·dL^−1^)	1.73 ± 0.32	0.83 ± 0.08^*∗*^	0.82 ± 0.04^*∗*^

*15-day treatment*			
Albumin (g·dL^−1^)	2.80 ± 0.16	2.52 ± 0.07	2.62 ± 0.08
ALT (U·L^−1^)	48.0 ± 7.60	48.8 ± 3.41	51.2 ± 4.27
AST (U·L^−1^)	80.2 ± 14.00	83.7 ± 12.04	81.1 ± 11.20
Cholesterol (mg·dL^−1^)	92.3 ± 18.90	97.4 ± 3.94	95.0 ± 4.48
Creatinine (mg·dL^−1^)	0.48 ± 0.03	0.92 ± 0.10^*∗∗*^	1.04 ± 0.10^*∗∗*^
GGT (U·L^−1^)	<10	<10	<10
Glucose (mg·dL^−1^)	89 ± 12.40	93 ± 6.67	122 ± 9.40
HDL (mg·dL^−1^)	54.4 ± 6.00	55.5 ± 2.53	55.4 ± 1.66
LDL (mg·dL^−1^)	28.5 ± 1.70	28.8 ± 1.65	22.8 ± 1.31
LDH (mg·dL^−1^)	357 ± 59.60	208 ± 43.20	349 ± 59.70
Triglycerides (mg·dL^−1^)	86.0 ± 3.51	55.0 ± 7.91^*∗*^	87.3 ± 6.94^‡^
Urea (mg·dL^−1^)	36.3 ± 1.49	30.4 ± 2.48	32.4 ± 1.12
Uric acid (mg·dL^−1^)	1.50 ± 0.25	1.52 ± 0.12	2.00 ± 0.28^*∗*^

*30-day treatment*			
Albumin (g·dL^−1^)	2.82 ± 0.02	2.66 ± 0.10	2.74 ± 0.07
ALT (U·L^−1^)	49.32 ± 10.04	47.1 ± 8.17	51.0 ± 9.13
AST (U·L^−1^)	83.0 ± 7.38	84.1 ± 9.90	85.3 ± 10.02
Cholesterol (mg·dL^−1^)	97.5 ± 0.65	90.0 ± 1.96^*∗*^	92.5 ± 3.43
Creatinine (mg·dL^−1^)	0.48 ± 0.02	0.70 ± 0.03^*∗∗*^	0.72 ± 0.02^*∗∗*^
GGT (U·L^−1^)	<10	<10	<10
Glucose (mg·dL^−1^)	74 ± 10.50	88 ± 10.80	90 ± 13.90
HDL (mg·dL^−1^)	56.2 ± 1.11	57.0 ± 3.70	57.0 ± 3.39
LDL (mg·dL^−1^)	26.3 ± 2.33	23.3 ± 2.73	28.8 ± 1.20
LDH (mg·dL^−1^)	322 ± 40.00	331 ± 90.70	371 ± 27.80
Triglycerides (mg·dL^−1^)	81.8 ± 5.02	56.8 ± 4.80^*∗∗*^	50.0 ± 3.91^*∗∗*^
Urea (mg·dL^−1^)	36.0 ± 1.38	34.6 ± 1.33	38.4 ± 1.29
Uric acid (mg·dL^−1^)	1.26 ± 0.87	0.74 ± 0.07^*∗∗*^	0.84 ± 0.09^*∗∗*^

ALT, alanine aminotransferase; AST, aspartate aminotransferase; EP, Específico-Pessôa phytotherapic tincture; GGT, gamma glutamyltransferase; HDL, high-density lipoprotein cholesterol; LDL, low-density lipoprotein cholesterol, LDH, lactate dehydrogenase. ALT, AST, and GGT values represent the mean ± SEM of 3 animals. The other parameters represent the mean ± SEM of 5 animals. ^‡^ *P* < 0.05 for the comparison between the EP and alcohol groups. ^*∗*^ *P* < 0.05, ^*∗∗*^ *P* < 0.01 for the comparison with the relevant control group. The *P* values represent Dunnett's test.

**Table 3:** Effect of the indicated treatments on hematological parameters in male rats.

Parameter	Control	EP	Alcohol
*10-day treatment*			
Hemoglobin (g/dL)	14.7 ± 0.70	15.4 ± 1.20	16.1 ± 0.21
Total red blood cells (10^6^/*μ*L)	8.2 ± 0.41	9.4 ± 0.80	9.1 ± 0.34
Total white blood cells (10^3^/*μ*L)	5.2 ± 0.36	5.4 ± 0.88	5.1 ± 0.70
Neutrophils (%)	25.6 ± 1.65	25.2 ± 2.18	25.3 ± 1.21
Lymphocytes (%)	71.0 ± 1.13	70.2 ± 2.34	72.0 ± 1.78
Eosinophils (%)	0.40 ± 0.28	0.60 ± 0.90	0.40 ± 0.88
Monocytes (%)	3.0 ± 0.43	3.8 ± 1.12	2.30 ± 0.31
Basophils (%)	0.0 ± 0.0	0.20 ± 0.20	0.0 ± 0.0
Packed cell volume (%)	47.2 ± 0.47	49.6 ± 2.10	48.6 ± 0.54
Mean corpuscular volume (fL)	58.0 ± 3.63	54.1 ± 4.90	53.9 ± 1.91
Mean corpuscular Hb (pg)	18.1 ± 1.49	17.0 ± 2.20	18.0 ± 0.57
Mean corpuscular Hb (%)	31.1 ± 1.52	31.4 ± 2.80	33.4 ± 0.12

*15-day treatment*			
Hemoglobin (g/dL)	16.1 ± 0.23	16.0 ± 0.20	16.5 ± 0.13
Total red blood cells (10^6^/*μ*L)	8.9 ± 0.04	7.6 ± 0.24	8.1 ± 0.98
Total white blood cells (10^3^/*μ*L)	4.8 ± 0.03	4.2 ± 0.41	5.5 ± 1.09
Neutrophils (%)	22.5 ± 3.51	23.2 ± 2.53	16.4 ± 6.48
Lymphocytes (%)	74.5 ± 3.51	72.8 ± 6.52	78.3 ± 2.31
Eosinophils (%)	0.0 ± 0.0	0.0 ± 0.0	0.0 ± 0.0
Monocytes (%)	3.0 ± 1.00	4.0 ± 0.72	5.30 ± 2.41
Basophils (%)	0.0 ± 0.0	0.0 ± 0.0	0.0 ± 0.0
Packed cell volume (%)	47.2 ± 0.62	46.4 ± 0.21	47.2 ± 1.00
Mean corpuscular volume (fL)	54.2 ± 0.04	59.9 ± 5.40	63.0 ± 1.31
Mean corpuscular Hb (pg)	18.6 ± 0.33	20.7 ± 1.87	21.6 ± 0.62
Mean corpuscular Hb (%)	34.3 ± 0.50	34.6 ± 0.23	34.3 ± 0.40

*30-day treatment*			
Hemoglobin (g/dL)	15.8 ± 0.11	16.1 ± 0.32	16.4 ± 0.09
Total red blood cells (10^6^/*μ*L)	7.4 ± 0.58	7.4 ± 0.31	7.5 ± 1.34
Total white blood cells (10^3^/*μ*L)	6.8 ± 0.38	5.7 ± 0.59	7.0 ± 0.71
Neutrophils (%)	33.2 ± 4.91	27.6 ± 3.52	22.6 ± 2.55
Lymphocytes (%)	64.0 ± 4.53	69.2 ± 4.56	75.8 ± 2.70
Eosinophils (%)	0.60 ± 0.60	0.20 ± 0.20	0.0 ± 0.0
Monocytes (%)	1.8 ± 1.03	2.8 ± 1.21	1.6 ± 0.48
Basophils (%)	0.4 ± 0.20	0.20 ± 0.20	0.0 ± 0.0
Packed cell volume (%)	45.4 ± 0.17	46.6 ± 0.68	48.0 ± 1.03
Mean corpuscular volume (fL)	64.0 ± 6.63	63.5 ± 2.45	63.8 ± 1.71
Mean corpuscular Hb (pg)	22.3 ± 2.34	21.9 ± 0.90	21.9 ± 0.27
Mean corpuscular Hb (%)	34.8 ± 0.32	34.5 ± 0.21	34.4 ± 0.62

EP, Específico-Pessôa phytotherapic tincture; Hb, hemoglobin. Values represent the mean ± SEM of 5 animals.
